# Everolimus safety and efficacy for renal angiomyolipomas associated with tuberous sclerosis complex: a Spanish expanded access trial

**DOI:** 10.1186/s13023-016-0517-9

**Published:** 2016-09-26

**Authors:** Nicolás Roberto Robles, Ramón Peces, Álvaro Gómez-Ferrer, Felipe Villacampa, Jose Luis Álvarez-Ossorio, Pedro Pérez-Segura, Juan Morote, Bernardo Herrera-Imbroda, Javier Nieto, Joaquín Carballido, Urbano Anido, Marian Valero, Cristina Meseguer, Roser Torra

**Affiliations:** 1 Department of Nephrology, Hospital Universitario Infanta Cristina, REDINREN, Avenida de Elvas, S/N, 06006 Badajoz, Spain; 2 Department of Nephrology, Hospital Universitario La Paz, REDINREN, Paseo de la Castellana, 261, 28046 Madrid, Spain; 3 Department of Urology, Instituto Valenciano de Oncología, Calle del Profesor Beltrán Bàguena, 8, 46009 Valencia, Spain; 4 Department of Urology, Hospital Universitario 12 de Octubre, Avenida de Córdoba, S/N, 28041 Madrid, Spain; 5 Department of Urology, Hospital Universitario Puerta del Mar, Avenida Ana Viya, 21, 11009 Cádiz, Spain; 6 Department of Oncology, Hospital Universitario Clínico San Carlos, Calle del Profesor Martín Lagos, S/N, 28040 Madrid, Spain; 7 Department of Urology, Hospital Universitario Vall d’Hebron, Paseo de la Vall d’Hebron, 119-129, 08035 Barcelona, Spain; 8 Department of Urology, Hospital Universitario Virgen de la Victoria, Campus de Teatinos, S/N, 29010 Málaga, Spain; 9 Department of Nephrology, Hospital General Universitario de Ciudad Real, Calle del Obispo Rafael Torija, S/N, 13005 Ciudad Real, Spain; 10 Department of Urology, Hospital Universitario Puerta de Hierro Majadahonda, Calle de Manuel de Falla, 1, 28222 Majadahonda, Spain; 11 Department of Oncology, Hospital Clínico Universitario de Santiago de Compostela, Travesía de Choupana, S/N, 15706 Santiago de Compostela, Spain; 12 Department of Medical Affairs, Novartis Farmacéutica S.A, Gran Via de les Corts Catalanes, 764, 08013 Barcelona, Spain; 13 Inherited Renal Diseases, Department of Nephrology, Fundació Puigvert, REDINREN, IIB Sant Pau, Universitat Autónoma de Barcelona, Cartagena, 340-350, 08025 Barcelona, Spain

**Keywords:** Angiomyolipoma, Everolimus, Safety, Tuberous sclerosis complex

## Abstract

**Background:**

Renal angiomyolipomas (AML) are usual manifestations of tuberous sclerosis complex (TSC) that may cause aneurism-related haemorrhages and renal impairment. Everolimus has emerged as an alternative to surgery/embolization. We provide further insight into everolimus safety and efficacy for TSC-related AML.

**Methods:**

This was a Spanish expanded access trial including patients aged ≥18 years with TSC-related AML. They received 10 mg everolimus once daily until AML progression, unacceptable toxicity, death/withdrawal, commercialisation for TSC-related AML, or 1 year after first patient enrolment. The primary outcome was dose-limiting safety according to grade 3/4 adverse events, serious adverse events, or adverse events leading to treatment modification. Secondary outcomes included overall safety and efficacy.

**Results:**

Nineteen patients were enrolled and received everolimus for a median of 6.6 (5.3–10.9) months. Eleven (57.9 %) remained on 10 mg/day throughout the study and eight (42.1 %) required treatment modifications due to adverse events; none permanently discontinued treatment. Adverse events were overall grade 1/2 and most frequently included aphthous stomatitis/mucosal inflammation, hypercholesterolaemia/hypertriglyceridaemia, urinary tract infection, hypertension, dermatitis acneiform, and insomnia. Four (21.1 %) patients experienced grade 3 adverse events, none was grade 4, and only one (5.3 %) was serious (pneumonia). AML volume was reduced ≥30 % in 11 (57.9 %) patients and ≥50 % in 9 (47.4 %); none progressed. Right and left kidney sizes decreased in 16 and 14 patients, respectively.

**Conclusions:**

These findings support the benefit of everolimus for renal AML due to a manageable safety profile accompanied by reduced AML and kidney volumes.

**Trial registration:**

EudraCT number 2012-005397-63; date of registration 22 Nov 2012.

## Background

Tuberous sclerosis complex (TSC) is an autosomal dominant genetic disorder usually caused by mutations in either the *TSC1* or *TSC2* genes [1, 2] and which affects one in about 8000 to 12,900 individuals [3, 4]. These mutations spearhead mammalian target of rapamycin (mTOR) activation, leading to uncontrolled cellular proliferation [5]. The disease is characterized by neurocognitive deficits and growth of non-malignant tumours called hamartomas in several body locations, including renal angiomyolipomas (AML) in up to 80 % of patients along with other manifestations [6]. These AML are mesenchymal tumours composed of abnormal blood vessels, immature smooth muscle cells and adipose tissue [7]. They usually appear in childhood and their progressive enlargement leads to a high risk of bleeding and may sometimes encroach on the renal parenchyma causing renal failure [7]. Unlike sporadic renal AML, TSC-related AML tends to be larger, multiple, and at higher risk of bleeding [8]. The main goals of treating patients with renal AML are preserving kidney function and preventing complications such as haemorrhages. Surgical procedures or embolization has been used to manage large, symptomatic, and/or bleeding AMLs, but mTOR inhibitors have now emerged as a non-invasive treatment alternative [9].

Everolimus is an orally bioavailable mTOR inhibitor that induces cell cycle arrest, reduces cell proliferation, and prompts angiogenesis regression, contributing to suppress the enlargement of tumours and promoting their regression [10, 11]. Everolimus has demonstrated clinical activity across a variety of tumours, leading to its approval for malignancies such as hormone receptor-positive advanced breast cancer, neuroendocrine tumours of pancreatic origin and renal cell carcinoma [12]. The EXIST-2 phase III trial investigated the use of everolimus for renal AML associated with TSC [13]. It was a randomized, double-blind, placebo-controlled trial that demonstrated the efficacy of everolimus administration to adult patients with TSC-related AML, showing that over half of patients experienced at least a 50 % reduction in AML volume after just 6 months of treatment [13]. The efficacy and manageable safety profile seen in this trial were consistent with those observed in its subsequent extension phase [14]. In addition, everolimus safety appeared to be similar to that previously reported in other TSC populations [15–18] and did not give rise to safety concerns with regard to its use for different solid tumours [11]. This positive benefit/risk balance supported the use of everolimus for TSC-related renal AML and was the basis for requesting the European Medicines Agency’s authorization for this indication.

In light of the above, we decided to conduct an expanded access trial to provide further insight into the safety and efficacy of everolimus for the management of renal AML associated with TSC in Spain.

## Methods

This trial was conducted in accordance with the World Medical Association Declaration of Helsinki, all its amendments, and national regulations. It was approved by the ethics committee of Fundació Puigvert (Barcelona, Spain) and all patients gave their written informed consent prior to any study procedure.

### Patient population

This study included all patients meeting selection criteria who agreed to participate and were consecutively recruited between May 2013 and May 2014. The main inclusion criteria comprised patients aged 18 years or older, with at least one renal AML of 3 cm or larger in its longest diameter as per computed tomography (CT) or magnetic resonance imaging (MRI), and a definite diagnosis of TSC. This diagnosis was based on the modified Gomez criteria [19, 20]. Patients whose AML required surgery at enrolment were excluded, as well as those with AML-related bleeding or embolisation during the 6 months prior to enrolment. Other exclusion criteria mainly included previous history of heart attack, angina pectoris, haemorrhagic stroke related to atherosclerosis, impaired lung functioning, organ transplantation, or any surgery within the 2 months prior to enrolment, and also the presence of significant haematological/hepatic abnormality, serum creatinine levels higher than 1.5 times the upper limit of normal, haemorrhagic diathesis or treatment with vitamin K antagonists (except for low-dose warfarin), uncontrolled hyperlipidaemia/diabetes, other uncontrolled/severe disease that might cause unacceptable safety risks, or any ongoing/active infection (except for hepatitis B/C virus infection) at study enrolment.

### Study design and treatment

**Fig. 1:**
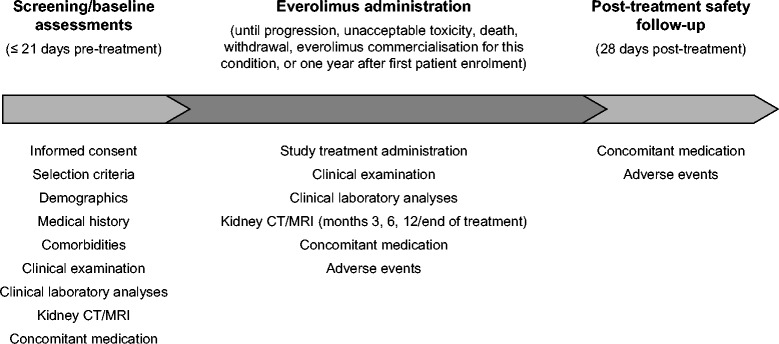
Overall flow chart and main study assessments. Abbreviations: *CT* computed tomography, *MRI* magnetic resonance imaging

This was an open-label, single-arm, phase IIIb, expanded access trial carried out at 12 Spanish hospitals. Screening and baseline assessments were conducted over the 21 days prior to the first dose of everolimus (Fig. 1). Eligible patients then started once-daily oral administration of everolimus at a dose of 10 mg/day after signing the informed consent. Everolimus was administered until AML progression, occurrence of unacceptable toxicity according to investigator’s criteria, patient death or withdrawal for any reason. Another criterion to stop the trial was everolimus commercialisation for TSC-related AML in Spain, or 1 year after first patient enrolment; the latter was what occurred first. Treatment modifications were determined clinically on the basis of safety findings (i.e. according to the grade of adverse events as per the Common Terminology Criteria for Adverse Events (CTCAE) of the National Cancer Institute version 4.03 [21]), including dose adjustments, temporary treatment interruptions or permanent treatment discontinuation. Initial doses of 10 mg/day could be lowered to 5 mg/day (dosing level −1) or even to 5 mg/every other day (dosing level −2).

Prophylactic antiviral treatment was recommended in patients with detectable hepatitis B virus DNA or surface antigen, although there were none. The use of antiproliferative agents or investigational drugs other than the study treatment was prohibited. Concomitant use of moderate/strong inhibitors or strong inducers of cytochrome P450 3A4 was to be avoided, as well as the use of p-glycoprotein inhibitors and attenuated vaccines. There were no further restrictions with regard to concomitant medication.

Toxicity of study treatment was continuously assessed according to adverse events reported in every monthly follow-up until 28 days post-treatment. All adverse events were coded using the Medical Dictionary for Regulatory Activities (MedDRA) preferred term and graded as per the CTCAE of the National Cancer Institute version 4.03 [21]. Their relationship with everolimus was categorized as non-related, unlikely, possibly, likely, or definitively related. AML response/progression was assessed by kidney CT/MRI. Local radiologists assessed measurable (longest diameter ≥1 cm) and non-measurable (longest diameter <1 cm) lesions at baseline, and at months 3, 6, and 12/end of treatment; for each patient, the same kidney imaging modality was used throughout the study. Radiologic response was defined as ≥50 % reduction from baseline in the sum of volumes of all target lesions (≤5 largest lesions with longest diameter ≥1 cm), confirmed in a second scan performed approximately 12 weeks later (no sooner than 8 weeks later), and in the absence of new lesions with longest diameter ≥1 cm, kidney volume increase >20 % from nadir, and AML-related grade ≥2 bleeding or need for embolisation/surgery. Radiologic progression was defined as ≥25 % increase from nadir in the sum of volumes of all target lesions and/or ≥20 % increase from nadir in the volume of either kidney with a value greater than baseline, appearance of new lesions with longest diameter ≥1 cm, and/or AML-related grade ≥2 bleeding or need for embolisation/surgery. Volume of each kidney was measured by CT/MRI to assess changes in non-target/non-measurable AML lesions.

### Statistical analysis

The primary outcome was dose-limiting safety according to the incidence of grade 3/4 adverse events, serious adverse events, and those leading to changes in the administration of everolimus (i.e. dose change, interruption, or discontinuation). Secondary outcomes included the overall safety of everolimus based on the incidence of any-grade adverse events and its efficacy according to the AML response rate. Descriptive analyses of patient characteristics and outcomes were conducted, including central tendency and dispersion (mean ± standard deviation [SD], or median [interquartile range, IQR]) for quantitative variables, and frequency distribution (absolute frequencies and valid percentages) for qualitative variables. Missing data were not considered in the analyses, which were performed with the Statistical Package for the Social Sciences version 19 (SPSS Inc, Chicago, USA) and R version 2.15.0 (The R Foundation, Vienna, Austria).

## Results

### Patient characteristics

A total of 20 patients were assessed for eligibility, one of whom was considered a screening failure as a result of suffering from an uncontrolled/severe disease that might have caused unacceptable safety risks. Therefore, 19 patients were finally enrolled and received the study treatment.

Their median (IQR) age was 38.0 (29.0–43.0) years and more than a half (68.4 %) were females (Table 1). They showed a median (IQR) sum of volumes of all target renal AML lesions of 260.0 (127.8–322.2) cm^3^; volumes of right and left kidneys reached 329.4 (193.0–979.7) cm^3^ and 299.0 (184.2–404.7) cm^3^, respectively.

**Table 1:** Baseline patient characteristics (*N* = 19)

Patient characteristics	Value
Age (years), median (IQR)	38.0 (29.0–43.0)
Sex, *n* (%)	
Female	13 (68.4)
Male	6 (31.6)
Race, *n* (%)	
White	19 (100)
Main comorbidities, *n* (%)^a^	
Hypertension	3 (15.8)
Asthma	2 (10.5)
Hypercholesterolaemia	2 (10.5)
Major features for TSC diagnosis, n (%)^a^	
Renal AML	19 (100)
Facial angiofibromas/forehead plaque	15 (79.0)
Cortical tuber	10 (52.6)
Hypomelanotic macules (≥3)	7 (36.8)
Subependymal nodule	7 (36.8)
Non-traumatic ungual/periungual fibroma	4 (21.1)
Single/multiple cardiac rhabdomyoma	4 (21.1)
Lymphangiomyomatosis	3 (15.8)
Connective tissue nevus	2 (10.5)
Minor features for TSC diagnosis, *n* (%)^a^	
Multiple renal cysts	11 (57.9)
Multiple randomly distributed pits in dental enamel	3 (15.8)
Bone cysts	3 (15.8)
Non-renal hamartoma	2 (10.5)
“Confetti” skin lesions	1 (5.26)
Sum of volumes of target renal AML lesions (cm^3^), median (IQR)	260.0 (127.8–322.2)
Volume of right kidney (cm^3^), median (IQR)	329.4 (193.0–979.7)^b^
Volume of left kidney (cm^3^), median (IQR)	299.0 (184.2–404.7)^c^

Abbreviations: *AML* angiomyolipoma, *IQL* interquartile range, *SD* standard deviation, *TSC* tuberous sclerosis complex

^a^Multiple response variable

^b^One patient had previously undergone nephrectomy of right kidney and two had technical problems for renal volume assessment; *N* = 16

^c^Three patients had previously undergone nephrectomy of left kidney and one had technical problems for renal volume assessment; *N* = 15

### Everolimus exposure

The median (IQR) duration of everolimus exposure was 6.6 (5.3–10.9) months. The weighted mean (±SD) dose was 9.1 ± 1.4 mg/day and the mean (±SD) accumulated dose reached 2123.0 ± 995.2 mg.

Eleven (57.9 %) patients received 10 mg/day of everolimus over the whole study duration. The remaining eight (42.1 %) patients required at least a dose reduction or temporary interruption of treatment. No patient permanently discontinued the study treatment.

### Safety outcomes

#### Primary safety outcome

A total of four (21.1 %) patients experienced five grade 3 adverse events, all of which were non-serious (Table 2). They included an increase in transaminases likely related to everolimus that led to the temporary interruption of treatment, hypertriglyceridaemia likely related to everolimus that required no therapeutic action, hypertriglyceridaemia definitively related to everolimus that required concomitant medication, hypertension likely related to everolimus that also required concomitant medication, and mucosal inflammation definitively related to everolimus that led to the temporary interruption of treatment. No grade 4 adverse event was reported.

**Table 2:** Dose-limiting safety (*N* = 19)

Outcome	*n* (%)
Patients with grade 3/4 adverse events	4 (21.1)^a^
Patients with serious adverse events	1 (5.3)^b^
Patients with adverse events leading to treatment modification:	8 (42.1)
Adverse events leading to dose reduction	3 (15.8)
Adverse events leading to temporary treatment interruption	3 (15.8)
Adverse events leading to dose reductions and temporary treatment interruption	2 (10.5)
Adverse events leading to permanent treatment withdrawal	0 (0.0)

All adverse events were graded as per the Common Terminology Criteria for Adverse Events (CTCAE) of the National Cancer Institute (version 4.03)

^a^Four patients experienced five grade 3 adverse events (i.e. transaminases increased *n* = 1, hypertriglyceridaemia *n* = 2, hypertension *n* = 1, mucosal inflammation *n* = 1); no grade 4 adverse event was reported

^b^The only serious adverse event was grade 2 pneumonia

Only one (5.3 %) patient reported a serious adverse event, which was a grade 2 pneumonia considered definitively related to everolimus and that led to the temporary interruption of treatment (Table 2).

Treatment modifications resulting from adverse events were reported in eight (42.1 %) patients: dose reductions in three (15.8 %), temporary treatment interruptions in another three (15.8 %), and both dose reduction and temporary treatment interruption in two (10.5 %) (Table 2). The reasons for dose reductions included menorrhagia, anaemia, hypercholesterolaemia, hypertriglyceridaemia, hypertension, and investigator’s decision due to accumulated adverse events rather than a specific event. The reasons for temporary treatment interruption encompassed gamma-glutamyltransferase increased, pneumonia, polypectomy, herpes zoster, mucosal inflammation, transaminases increased, and erythema.

#### Secondary safety outcome

All patients showed at least one of the 158 reported adverse events, regardless of relationship to study drug (adverse events of any cause). They were mostly grade 1/2 in severity and most frequently included (>25 % of patients): aphthous stomatitis, hypercholesterolaemia, hypertriglyceridaemia, urinary tract infection, mucosal inflammation, hypertension, dermatitis acneiform, and insomnia (Table 3).

**Table 3:** Adverse events of any cause experienced by ≥10 % patients over the study (*N* = 19)

Adverse event, *n* (%)	All grades	Grade 1	Grade 2	Grade 3
Aphthous stomatitis	11 (57.9)	11 (57.9)	0 (0.0)	0 (0.0)
Hypercholesterolaemia	11 (57.9)	5 (26.3)	6 (31.6)	0 (0.0)
Hypertriglyceridaemia	8 (42.1)	3 (15.8)	3 (15.8)	2 (10.5)
Urinary tract infection	8 (42.1)	2 (10.5)	6 (31.6)	0 (0.0)
Mucosal inflammation	7 (36.8)	5 (26.3)	1 (5.3)	1 (5.3)
Hypertension	6 (31.6)	3 (15.8)	2 (10.5)	1 (5.3)
Dermatitis acneiform	5 (26.3)	5 (26.3)	0 (0.0)	0 (0.0)
Insomnia	5 (26.3)	5 (26.3)	0 (0.0)	0 (0.0)
Catarrh	4 (21.1)	4 (21.1)	0 (0.0)	0 (0.0)
Diarrhoea	4 (21.1)	3 (15.8)	1 (5.3)	0 (0.0)
Amenorrhoea	3 (15.8)	3 (15.8)	0 (0.0)	0 (0.0)
Asthenia	3 (15.8)	3 (15.8)	0 (0.0)	0 (0.0)
Headache	3 (15.8)	3 (15.8)	0 (0.0)	0 (0.0)
Dysgeusia	3 (15.8)	3 (15.8)	0 (0.0)	0 (0.0)
Pharyngitis	3 (15.8)	3 (15.8)	0 (0.0)	0 (0.0)
Nasopharyngitis	3 (15.8)	2 (10.5)	1 (5.3)	0 (0.0)
Cough	3 (15.8)	3 (15.8)	0 (0.0)	0 (0.0)
Menstrual disorder	3 (15.8)	3 (15.8)	0 (0.0)	0 (0.0)
Conjunctivitis	2 (10.5)	2 (10.5)	0 (0.0)	0 (0.0)
Dermatitis	2 (10.5)	2 (10.5)	0 (0.0)	0 (0.0)
Back pain	2 (10.5)	2 (10.5)	0 (0.0)	0 (0.0)
Abdominal pain upper	2 (10.5)	2 (10.5)	0 (0.0)	0 (0.0)
Epistaxis	2 (10.5)	2 (10.5)	0 (0.0)	0 (0.0)
Erythema	2 (10.5)	1 (5.3)	1 (5.3)	0 (0.0)
Rash	2 (10.5)	2 (10.5)	0 (0.0)	0 (0.0)
Oral herpes	2 (10.5)	2 (10.5)	0 (0.0)	0 (0.0)
Dizziness	2 (10.5)	2 (10.5)	0 (0.0)	0 (0.0)
Menorrhagia	2 (10.5)	2 (10.5)	0 (0.0)	0 (0.0)
Myalgia	2 (10.5)	2 (10.5)	0 (0.0)	0 (0.0)
Pyrexia	2 (10.5)	2 (10.5)	0 (0.0)	0 (0.0)
Vomiting	2 (10.5)	2 (10.5)	0 (0.0)	0 (0.0)
Gamma-glutamyltransferase increased	2 (10.5)	0 (0.0)	2 (10.5)	0 (0.0)
Transaminases increased	2 (10.5)	0 (0.0)	1 (5.3)	1 (5.3)

All adverse events were coded using the Medical Dictionary for Regulatory Activities (MedDRA) preferred term and graded as per the Common Terminology Criteria for Adverse Events (CTCAE) of the National Cancer Institute (version 4.03)

Overall, 18 (94.7 %) patients reported 106 adverse events suspected to be everolimus related (i.e. possibly, likely, or definitively related). They were mostly grade 1/2 and most commonly included (>25 % of patients): aphthous stomatitis (*n* = 11, 57.9 %), hypercholesterolaemia (*n* = 11; 57.9 %, grade 1 in five patients and grade 2 in six patients), hypertriglyceridaemia (*n* = 8; 42.1 %), urinary tract infection (*n* = 6; 31.6 %), mucosal inflammation (*n* = 7; 36.8 %), and dermatitis acneiform (*n* = 5; 26.3 %).

A total of 45 infections were observed in 14 (73.7 %) patients, which were grade 1/2 and affected the oral cavity (*n* = 16), respiratory tract (*n* = 12), urinary tract (*n* = 8), skin (*n* = 7), and eyes (*n* = 2). Only two renal adverse events were reported, including grade 1 proteinuria (i.e. 1+ proteinuria; urinary protein <1.0 g/24 h) (*n* = 1) and polyuria (*n* = 1); neither haemorrhage of renal AML nor increase of creatinine levels was reported. Amenorrhoea was reported in three patients, which accounted for 27.3 % of 11 pre-menopausal females and was grade 1 in all cases (i.e. intermittent menses with skipped menses for no more than 1 to 3 months); it was recovered in two patients and remained ongoing at the end of the study in another, who developed it 1 month before the study completion. Regarding hypercholesterolaemia: no action was taken in two patients (grade 1), there was no study treatment interruption/discontinuation in any patient, and a concomitant treatment was reported in nine patients.

### Efficacy outcomes

Nine (47.4 %) patients reported radiologic response of renal AML, with a median (IQR) time from everolimus initiation to the response assessment of 3.3 (3.0–6.2) months. Ten (52.6 %) patients remained stable (i.e. neither radiologic response nor progression) and none showed AML progression.

**Fig. 2:**
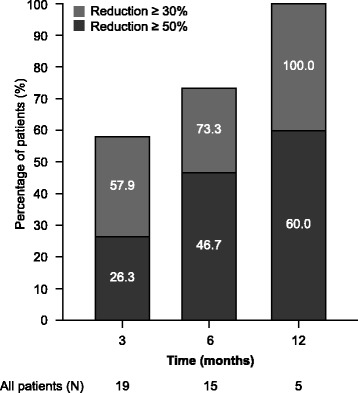
Reductions from baseline in renal angiomyolipoma volume in each study visit. The study ended as per protocol 1 year after first patient enrolment, which explains the reduced number of patients available at month 12

The sum of volumes of all target AML lesions decreased in 16 (84.2 %) patients, including ≥30 % reduction from baseline in 11 (57.9 %) patients and ≥50 % in nine (47.4 %) patients; the proportion of patients achieving ≥30 % and ≥50 % reduction from baseline in each study visit is summarized in Fig. 2. Only three (15.8 %) patients report increased volumes, which were lower than 15 % in all cases.

The volume of right kidney was reduced in 16 patients; no data was available on right kidney volume in three patients (two due to technical problems and one due to nephrectomy). The volume of left kidney decreased in 14 patients and only one patient showed increased left kidney volume, which was lower than 20 %; no data was available in four patients (one due to technical problems and three due to nephrectomy).

## Discussion

This phase IIIb expanded access trial supports the benefit of everolimus administration to adult patients with TSC-related renal AML, due to a manageable safety profile accompanied by reduced AML lesions and kidney volumes. The overall safety profile was as expected according to previously described adverse events experienced by patients with TSC during everolimus administration [13–18]. Oral mucosal inflammation with/without aphthae, hypercholesterolaemia, hypertriglyceridaemia and infections such as those of the urinary tract were reported among the most frequent adverse events. These were mostly mild to moderate and could be appropriately managed without everolimus discontinuation, with decreased dose and/or adjunctive therapy. Indeed, initial everolimus administration was maintained in nearly 60 % of patients and it could be continued in the remaining patients after dose reductions and/or transient interruptions. Although the occurrence of grade 3/4 adverse events might have jeopardized the administration of everolimus, only five grade 3 and no grade 4 events were reported over the study duration and most of them could be appropriately managed with concomitant medication or just observation; only two required temporary treatment interruptions. Furthermore, only one patient reported a serious adverse event, a case of pneumonia that also led to temporary treatment interruption. These results are consistent with data reported by the EXIST-2 trial on the administration of everolimus to adult patients with TSC-related AML [13, 14] and other trials including the EXIST-1 on everolimus administration to adult/child patients with other TSC-related tumours [15–18]. Adverse events have usually been reported in these trials in a considerable percentage of patients over the first year of administration; however, they were mostly grade 1/2 in severity and few were serious. In addition, adverse events were usually handled with concomitant medications and/or dose reductions/interruptions, this making treatment discontinuation an infrequent need. Inflammation of the mucous membranes of the oral cavity with/without the formation of oral ulcerations are usual and potentially dose-limiting events during everolimus administration that may appear in 70 to 100 % of patients receiving this agent for TSC-related tumours [13–18]. In addition, the immunosuppressive properties of everolimus may also increase susceptibility to localized or systemic infections, which may occur in 65 to 100 % patients [13–18]; however, clinical and therapeutic drug monitoring of patients under everolimus treatment may considerably limit its consequences and improve patients’ safety. The minimum dose necessary to maintain target everolimus plasma levels may avoid over treatment and therefore may limit some adverse events. Previously conducted studies have reported the occurrence of certain adverse events such as infections not only in patients receiving everolimus but also in those receiving placebo [13–16], which raises the issue of whether there is a predisposing effect of the disease that needs to be clarified in future studies. Metabolic events such as hypercholesterolaemia, hypertriglyceridaemia are other potential risks associated with everolimus treatment that have been reported in 11 to 42 % of patients [13, 14, 17, 18], which may require corrective measurements including dietary adjustments and/or cholesterol lowering medication to prevent any potential long-term cardiovascular effects. Furthermore, amenorrhoea has recently emerged as a potential adverse event affecting 13 to 38 % at-risk females during everolimus administration [13–16]. Although its relationship to everolimus is undergoing investigation, it reinforces the need for further surveillance in women of childbearing potential, though pregnancy must be avoided while on treatment. Our female population was two-thirds of the overall population, most of which were pre-menopausal, and though yet the prevalence of AML in females and males in TSC seems to be equal, it should be clarified if females have an increased risk of AML. Furthermore, despite the fact that only one patient reported proteinuria, a more systematic assessment of proteinuria/albuminuria should be considered in future studies to confirm our findings.

Everolimus administration to patients with TSC-related AML over the extension phase of the EXIST-2 trial supports a long-term adverse event profile consistent with previously established risks and with no new safety concerns [14]. In addition, the incidence of adverse events markedly decreased over time, with the highest incidence over the first year of treatment, then declining and reaching rates lower than 10 % in most adverse events over the second year and even lower over the third year [14]. The decreasing incidence of adverse events and the fact that most of them could be managed successfully through dose reductions/interruptions support the importance of careful monitoring and effective management to optimize patient safety and treatment outcomes. An acceptable safety profile is absolutely needed in this setting, as young adults may be treated lifelong and increased cardiovascular risk should be avoided.

Continued exposure to everolimus has shown to provide a sustained reduction of TSC-related renal AML lesions over time [14]. These effects may become evident a few months after starting therapy, with a median time to AML response for everolimus of nearly 3 months [13, 14]. The rapid clinical activity of everolimus was especially important in our patient population, which showed large AMLs and were therefore at risk of complications. Indeed, most patients achieved benefits in terms of reducing renal AML lesions and kidney volumes after a median of 3.3 months of therapy, without exhibiting AML-related complications such as haemorrhages or deterioration of renal functioning over the whole study follow-up. These data are in line with the renal AML shrinkage shown by previous trials and support the achievement of greater benefits after longer administration periods [13, 14], which may even reach up to 86.4 % of patients achieving AML reductions ≥30 % over the first 3 years of treatment [14]. This AML reduction also supports the role of everolimus as a therapeutic alternative to traditional therapies such as selective arterial embolization, which often lead patients to need further embolisations or even nephrectomy in the long-term [22]. In addition, AML shrinkage over everolimus administration appears to occur irrespective of age, gender, and race [13, 16], and levels of plasma angiogenic markers such as endothelial growth factor D or collagen type IV seem to be associated with response to everolimus [23]. However, further studies are still needed to assess other patient characteristics and/or biomarkers that enable the magnitude of response to be optimized when tailoring treatment to patients with TSC-related renal AMLs, as well as determining the most appropriate treatment duration to maximize and prolong everolimus’ effect over time.

The safety and efficacy of everolimus administration for TSC-related AML evidenced in our trial is in line with our previous experience with sirolimus use in this condition. The findings derived from our previous 24-month phase II-III study carried out in 17 patients with TSC-related AML also evidenced an acceptable safety profile of sirolimus, with stomatitis and hypertriglyceridaemia as the main adverse reactions [24]. The mTOR inhibition caused by sirolimus was effective in reducing AML volume, with a faster shrinkage at the beginning of treatment that was likely related to its anti-angiogenic effect. These results support the role of mTOR inhibitors as promising treatment alternatives for TSC-related AML, with a manageable safety profile, adequate efficacy and less aggressiveness than other therapeutic options currently available.

The authors acknowledge that the present trial has several limitations that should be considered when interpreting its findings, including the open-label and single-arm design. Although the absence of a control group prevented us from determining the magnitude of the effect, the favourable data available on everolimus risk/benefit balance would have made the use of placebo unethical and there was no alternative non-surgical standard therapy for the disease. Local radiologists were involved in all CT/MRI assessments and no independent central radiologic review was conducted, which may have increased inter-observer variability. Furthermore, this study could only address the short-term effect of everolimus administration and the reduced sample size of our study can be regarded as another limitation. Nonetheless, our findings expand the current information on the use of everolimus for this relatively uncommon condition.

## Conclusion

This trial supports the safety profile and clinical efficacy of everolimus for the treatment of TSC-related AML reported by previous clinical trials. Adverse events exhibited over the study were as expected, without raising new safety concerns, and were successfully managed with concomitant medication, dose reductions, and/or temporary treatment interruptions. This manageable safety profile enabled patients to remain under treatment with everolimus, achieving benefits in terms of AML lesions and kidney volumes. Reductions in these AML lesions and kidney volumes were evident in the majority of patients a few months after starting treatment, more than half achieved radiologic AML response, and none progressed. These findings expand the information currently available on the use of everolimus for this condition and warrant further assessments to optimize and prolong everolimus’ effect in routine clinical practice.

## Abbreviations

AML: Angiomyolipoma
CT: Computed tomography
CTCAE: Common Terminology Criteria for Adverse Events
IQL: Interquartile range
MedDRA: Medical Dictionary for Regulatory Activities
MRI: Magnetic resonance imaging
mTOR: Mammalian target of rapamycin
SD: Standard deviation
TSC: Tuberous sclerosis complex
